# The Brainstem-Informed Autism Framework: Early Life Neurobehavioral Markers

**DOI:** 10.3389/fnint.2021.759614

**Published:** 2021-11-10

**Authors:** Or Burstein, Ronny Geva

**Affiliations:** ^1^Department of Psychology, Bar-Ilan University, Ramat Gan, Israel; ^2^Gonda Multidisciplinary Brain Research Center, Bar-Ilan University, Ramat Gan, Israel

**Keywords:** autism spectrum disorders (ASD), brainstem, auditory brainstem evoked response (ABR), respiratory sinus arrhythmia (RSA), sleep, sensory processing, arousal, neonates

## Abstract

Autism spectrum disorders (ASD) have long-term implications on functioning at multiple levels. In this perspective, we offer a brainstem-informed autism framework (BIAF) that traces the protracted neurobehavioral manifestations of ASD to early life brainstem dysfunctions. Early life brainstem-mediated markers involving functions of autonomic/arousal regulation, sleep-wake homeostasis, and sensorimotor integration are delineated. Their possible contributions to the early identification of susceptible infants are discussed. We suggest that the BIAF expands our multidimensional understanding of ASD by focusing on the early involvement of brainstem systems. Importantly, we propose an integrated BIAF screener that brings about the prospect of a sensitive and reliable early life diagnostic scheme for weighing the risk for ASD. The BIAF screener could provide clinicians substantial gains in the future and may carve customized interventions long before the current DSM ASD phenotype is manifested using dyadic co-regulation of brainstem-informed autism markers.

## Introduction

The brainstem and its rostral networks underlie a wide array of operations, ranging from autonomic functions such as respiration (Bianchi and Gestreau, 2009), cardiovascular activity (Dampney, 2016), and sleep-wake regulation (Scammell et al., 2017), through sensorimotor reactivity (Kobayashi and Isa, 2002), and even involvement in consciousness and self-awareness (Parvizi and Damasio, 2001).

Autism spectrum disorders (ASD) are a set of neurodevelopmental disorders manifested in deficits in social-communication abilities and restrictive and repetitive behaviors (American Psychiatric Association [APA], 2013). Despite the DSM nosology that classifies ASD as a unified construct, various findings suggest a high degree of heterogeneity in ASD phenomenological manifestation and genetic basis that nevertheless share common cellular and molecular features, including alterations in neurogenesis, synaptogenesis, and structural formation (Gilbert and Man, 2017). Recent accounts, some from our lab, emphasize the role of early brainstem functions in the epiphenomena of ASD (Dadalko and Travers, 2018; Delafield-Butt and Trevarthen, 2018); namely, in social attention (Geva et al., 2017), communication (Geva et al., 2013, 2014), and repetitive behaviors (Cohen et al., 2013; Gandhi and Lee, 2021)– all key features of ASD.

Social attention, communication, and adaptation of behavior have primary roles in the human core being already at birth. Hence, we suggest that brainstem circuits that mature in very early life and even during fetal stages to regulate vital autonomic functions (Zec and Kinney, 2003), have a central role in shaping social communication and adaptation of behavior. We suggest that given the early maturation of brainstem pathways, their pervasive role in functioning at multiple levels, and their specific involvement in social-communication deficits, brainstem functions enable a valuable window into the pathophysiology of ASD. Its neurobehavioral manifestations are already evident in the first phases of postnatal life.

Research thus far suggests brainstem involvement in ASD by typically denoting a unitary brainstem marker. Building upon an integration of the literature, in the current perspective, we propose a brainstem-informed autism framework (BIAF) that zooms in on the distinct paths by which compromised brainstem functions possibly stir development and increase the susceptibility for ASD-related symptomatology. We then suggest that zooming out to look at the full battery of brainstem-related expressions, rather than individual markers, may enable constructing a highly sensitive early risk neurobehavioral screening tool of ASD.

## The Formation of Brainstem Networks

Principal morphological changes in the embryonic brainstem in multiple organisms buds during the first trimester of pregnancy (ten Donkelaar et al., 2014). Animal models indicate that the genesis of motoneurons in rhombomeres 7 and 8 commence approximately at the fourth week of fetal life; these neurons subsequently migrate and form the vagal nerve nuclei, including the dorsal motor nucleus, nucleus ambiguus, solitary tract nucleus, and spinal trigeminal nucleus (ten Donkelaar et al., 2014; Watson et al., 2019). The neural functionality of brainstem pathways is noted from early gestational stages (Glover et al., 2008; Marrs and Spirou, 2012). A post-mortem specimens study of the medulla in human fetuses indicated that a neural branching from and into the solitary tract nucleus is established and expedites cardiorespiratory control around gestational age (GA) of 20 weeks (Zec and Kinney, 2003). The development of vital parasympathetic functions is further secured from mid-gestation to parturition as myelination of the vagal nerve roots progresses (Tanaka et al., 1995). Importantly, myelination of efferent fibers from the nucleus ambiguus to the sinus nodes that regulate cardiac pace is accelerated (Porges, 2011) and stabilizes the parasympathetic activity when reaching term age as manifested by increased heart rate variability at the higher (*i.e.*, above 0.2 Hz) frequencies (Longin et al., 2006). Similar neuro-maturational processes involving the birth of neurons in hindbrain rhombomeres and mesencephalic neuromeres, neurons migration, and axonal navigation contribute to the formation of the cranial nerves sensorimotor nuclei in the brainstem from Carnegie stage 12 (O’Rahilly and Müller, 2006; ten Donkelaar et al., 2014). These structures support auditory, ocular, tactile, gustatory, and olfaction development and shape motor reactivity in a progressive fashion. As such, early postnatal myelination of axons radically increases the rate and synchronicity of transmission through the auditory pathway, emanating from the cochlear nuclei, superior olive, lateral lemniscus, and inferior colliculus (Sano et al., 2007), alongside other sensorimotor paths that evolve in tight temporospatial constraints.

Optimal structuring of the brainstem has vast implications on neurocognitive sequelae, as the early structural building blocks of these early maturing networks influence the emerging operations of higher-order top-down limbic and neocortical systems. Eventually, brainstem networks affect functions from basic reception through multisensory and motor integration (Geva and Feldman, 2008), in ways that affect behavioral inhibition (Geva et al., 2014), higher-order social engagement (Geva et al., 2017), and social communication capacities (Geva et al., 2013). As such, evaluation of brainstem integrity offers multiple candidate markers for ASD. These markers are potentially diagnosable at term age and soon thereafter.

To date, the research has mostly treated each BIAF factor as a single primary marker. We review each one shortly and suggest that their integration presents a strong case for a cohesive BIAF. We shall focus on the hallmarks of brainstem functions: cardiac, respiratory, and arousal regulation; sleep-wake homeostasis; and primary sensorimotor operations. Following the exploration of their main effects and interactions, we will delineate a BIAF, focusing on how it first unfolds in gestation and the post-birth period.

## Control of Arousal: Cardiovascular and Respiratory Development

The vertical hierarchical framework was formulated in our lab to delineate the development of self-regulation and positioned brainstem functions at the crux of the model (Geva and Feldman, 2008). According to this model, brainstem networks serve pivotal roles in regulating the young infant’s arousal responses to sensations. Recent notions accentuate that nascent autonomic conditioning of socioemotional reflexes and arousal responses occur at the brainstem and peripheral levels and prior to the top-down cortical navigation of arousal (Ludwig and Welch, 2020). Poor arousal regulation is one of the key features of ASD (Prince et al., 2017; Cuve et al., 2018; Corbett et al., 2019; de Vries et al., 2021), evident by hyper- or hypo-arousal reactivity of the autonomic system in response to sensory stimulation. Particularly in infants who are siblings of children with ASD (Zivan et al., 2021). The functional implications of autonomic dysfunctions in individuals with ASD are vividly apparent in an array of markers, including pupil diameter (Zivan et al., 2021), electrodermal activity (Prince et al., 2017), and the coordination of heart rate and breathing (Corbett et al., 2019). These autonomic functions are by and large regulated at first by brainstem networks that mature in late-term stages and have been suggested to serve social engagement purposes (Porges, 2001).

The inner workings of the mechanisms involved in this interplay are now under investigation using different models and an array of techniques. Here we note some of the literature that exemplifies the richness of data and intricate set of interlinked neurophysiological processes currently explored.

Possible causes for the abnormalities in autonomic functions in ASD are aberrations in cerebellar-brainstem white matter tracts, involving insufficient glial maturation and axonal growth differences noted in infancy and early childhood (Shukla et al., 2010; Yu et al., 2020), altered white matter connectivity of brainstem tracts found in tractography machine learning analysis (Zhang et al., 2018), and atypical structuring of the medullary arcuate nucleus which is involved in cardiorespiratory regulation (Bailey et al., 1998). The exact trajectory and localization of histogenesis and primal autonomic circuitries development in ASD remain to be further elucidated. Hopefully, future studies will clarify whether abnormal patterns of myelination, axonal navigation, and circuits formation of vagal nerve nuclei during embryonic and neonatal development are implicated in the autonomic sequelae of individuals with ASD. Even though the developmental pathophysiological course is not yet fully established, several cardiorespiratory indices were utilized to weigh the involvement of vagal functions in ASD research. We shall focus on respiratory sinus arrhythmia (RSA).

Respiratory sinus arrhythmia measures the variations in heart rate as a function of the respiration cycle and is regarded as an applicable index of the vagal tone and its coordination by the nucleus ambiguus (Berntson et al., 1993). Porges’ polyvagal theory proposes that inner physiological experiences are innervated by socio-emotional sensations right from birth and that this interplay underlies the nascent steps of social development (Porges, 1995, 2011, 2021).

A recent comprehensive meta-analysis (Cheng et al., 2020) involving participants with ages spanning the first three decades of life revealed that diagnosis of ASD was associated with diminished baseline RSA and diminished RSA reactivity during social experiences. A previous prospective study including a cohort of very preterm children has shown that neonatal RSA indices predicted social competence at the age of three (Doussard-Roosevelt et al., 1997) and then at school age (Doussard-Roosevelt et al., 2001). Further, infants diagnosed with ASD in late childhood showed a blunted pattern of RSA development from the age of 18 months (Sheinkopf et al., 2019). The RSA findings imply that the alignment of vagal resources with the social environment, mostly those involving the adaptive switching between tranquil/non-engaged and charged/engaged states, scaffolds the building blocks of social development from birth. It further accentuates that cardiovascular hypo- and hyper-arousal reactivity might affect vigilance and impede the prospect of a durable engagement with parents, peers, and significant others in ASD.

Vigilance models have been proposed to explain a range of psychopathological processes from mania to attention deficits (Hegerl and Hensch, 2014). These models have noted links between poor arousal regulation, unstable vigilance, and sleep deficits. We suggest that these notions are highly relevant to the BIAF.

## Sleep as a Social Awakener

Primal sleep-wake substrates in the brainstem promote sleep rhythms long before the anterior limbic circuits gain dominance (Villablanca et al., 2001). Given the primary involvement of brainstem networks in sleep regulation, the BIAF suggests that congenital compromised brainstem functions could instigate sleep-wake dysregulations from the neonatal period. Then, it might perturb the brainstem-limbo-cortical connectivity and lead to long-term sleep deficits (Geva and Feldman, 2010; Blumberg et al., 2014).

The primal sleep-wake system consists of wake-promoting loci in the reticular formation along the brainstem, including the monoaminergic locus coeruleus (LC) and dorsal raphe nucleus (DRN), and the cholinergic laterodorsal tegmental nucleus and parabrachial nuclei; the primal GABAergic sleep-promoting structures include the nucleus pontis oralis, nucleus subcoeruleus and the Purkinje cells in the cerebellum (Phillips and Robinson, 2007; Blumberg et al., 2014; Sokoloff et al., 2015). These brainstem-cerebellar hubs are highly implicated in the ultradian cyclicity of sleep-wake bouts during the first weeks of extrauterine life (Geva and Feldman, 2010). Infant sleep is marked by high rates of REM sleep that have a vital neuroprotective role and is guided by the aforementioned brainstem loci (Heraghty et al., 2008). Accordingly, lesions and morphological abnormalities in both gray and white matter in pontine and adjacent regions are associated with reduced REM sleep in human adults (Landau et al., 2005; Scherfler et al., 2011). Further, sleep organization and a smooth transition between sleep stages seem important. Fragmented neonatal sleep has been noted to impede infant attention orienting at 18 months of age (Geva et al., 2016). One should keep in mind these phenomena when considering the trajectories of sleep integrity in populations with ASD.

Children, adolescents, and adults with ASD display various sleep abnormalities, including decreased sleeping time, delayed sleep latency, and less efficient sleep (Elrod and Hood, 2015; Lugo et al., 2020; Chen et al., 2021), and show a strikingly elevated risk for sleep disorders (Lai et al., 2019; Lugo et al., 2020). These findings suggest that sleep deficits and ASD possibly stem from a similar neuropathological disrupted circuitry that involves the brainstem in a major way.

Studies have illustrated several influences of the brainstem in sleep-wake dysregulation in ASD populations. Vis-à-vis inhibitory pathways, genetic findings suggest that ASD is associated with microduplications copy-number variations in the chromosome 15q11–q13 region responsible for coding the GABA_*A*_ receptor’s subunits (Sebat et al., 2007; Meguro-Horike et al., 2011; Sanders et al., 2012). Accordingly, post-mortem studies found a decrease in GABAergic Purkinje neurons in cerebellum specimens of deceased ASD patients (Bailey et al., 1998; Palmen et al., 2004). Functional alterations in the activity of excitatory networks involving the LC are found in children with ASD (Bast et al., 2018; Huang et al., 2021). Taken together, these suggest that an imbalance between the primary excitatory (*e.g.*, the monoaminergic LC and DRN) and inhibitory (*e.g.*, the GABAergic Purkinje cells) circuits (Coghlan et al., 2012) of the brainstem is involved in the pathogenesis of sleep disturbances in ASD patients, as well as affecting other arousal-related domains (Wintler et al., 2020).

The extent of sleep deficits could also be viewed as a distinct factor that affects social development. Several findings support this latter notion. First, abnormal sleep patterns in early life are associated with subsequent ASD diagnosis and symptoms (Humphreys et al., 2014; Saenz et al., 2015; Miike et al., 2020). Further, in children with ASD, shorter sleep duration exacerbates the severity of both repetitive behaviors and social-communication deficits (Schreck et al., 2004; Tudor et al., 2012; Veatch et al., 2017). The hazards posed by sleep disruptions in children with ASD urged clinicians to recommend that sleep-wake homeostasis issues be assessed and managed as a central feature in the therapeutic plan of ASD patients (Cohen et al., 2014; Abel et al., 2017; Souders et al., 2017). This agenda accentuates the primary role sleep possibly serves in the evolution of ASD and the neuroprotective role of sleep in its containment (Wintler et al., 2020).

The findings suggest that sleep and arousal play a major role in ASD. Sleep disturbances are plausibly a result of fetal, genetic, and epigenetic brainstem-mediated antecedents. At the same time, they stand by themselves as factors that might exacerbate the risk for ASD or lead to more severe symptoms by impeding brain development and its regulated reactivity to stimulation through the various senses.

## The Stem of the Senses

Atypical behavioral responses to sensory stimulation are a ubiquitous characteristic of ASD (Marco et al., 2011). Research on unimodal sensory processing and multisensory integration using various neuroimaging techniques demonstrated significant alterations in sensory processing neural substrates (Marco et al., 2011). Here we briefly review some of the unimodal and multisensory processing findings that pertain to the BIAF.

### Auditory Processing

A feasible aperture into neonatal brainstem auditory functions involves the highly utilized auditory brainstem evoked response (ABR) test. The ABR is broadly implemented across the globe as a screener for hearing deficits in newborns (Morton and Nance, 2006; Levit et al., 2015). Diving into its characteristics enables uncovering its germaneness to autism. In the ABR procedure, newborns are exposed to auditory stimulations (*i.e.*, click or speech sounds in standardized dB levels) while an electrode attached to the scalp measures the electrophysiological activity. The latencies of neuro-electrical fluctuations following the auditory stimuli are typically manifested in five major wave peaks in neuro-typical adults; waves I and II originate from the auditory vestibular nerve, while wave peaks III–V putatively reflect the reaction of deeper structures including the cochlear nuclei (wave III), superior olive (wave IV) and lateral lemniscus and inferior colliculus (wave V; Wilkinson and Jiang, 2006). Apart from its conventional role for detecting hearing deficits, the ABR presents intriguing information on deficient brainstem maturation, detectable already in the late-term period in ways pertaining to ASD risk detection.

Both functional and structural brainstem auditory path alterations are noted in individuals with ASD. Changes in neural transmission rates through the brainstem (most prominently a delayed V peak latency) that appear already at birth have been associated with increased risk for subsequent diagnosis of ASD (Cohen et al., 2013; Miron et al., 2016, 2021; Tu et al., 2020). This association persists throughout infancy, toddlerhood, and childhood (Miron et al., 2018; Talge et al., 2018). Along with the functional differences in brainstem auditory structures, morphological studies have demonstrated durable alterations in size, volume, and neuronal density in the superior olive (Kulesza et al., 2011; Mansour and Kulesza, 2020), as well as abnormal geometric arrangements of cells body shape and orientation (Kulesza and Mangunay, 2008). Taken together, abnormalities in brainstem auditory centers may be a relatively stable marker of ASD, which stands so a long way before autism symptoms onset.

What does a deficient ABR at birth imply concerning the mechanisms driving ASD? Two non-mutually exclusive options come to mind: First, a deficit in reception, filtering, and processing of auditory signals (Morton and Nance, 2006; Levit et al., 2015). A deficit in auditory processing may account for persistent disruptions in neuro-cognitive development. Inability to perceive vocal cues and produce them well has a profound effect on social and communication capacities (Del Zoppo et al., 2015; Petersen and Hurley, 2017). Accordingly, an auditory processing deficit is highly prevalent in populations diagnosed with ASD (O’Connor, 2012; Williams et al., 2020). Impairments such as auditory hypersensitivity (Williams et al., 2021) and diminished background noise filtering (Park et al., 2017) can obstruct the ability to prepare ahead of time and might lead to intensified anxiety, repetitive behaviors, and a strong need to keep routines and rigidly anticipated schedules (Schaaf et al., 2011; Kargas et al., 2015; Kanakri et al., 2017; Park et al., 2017; Ahmmed and Mukherjee, 2021). As such, the auditory path alone already accounts well for a large portion of ASD phenomenology.

Alternatively, even when the infant’s hearing threshold is preserved, an asynchronous auditory nerve firing or delayed processing evident in the ABR may signal altered neural programming that operates in a pervasive manner. These alterations affect a widely distributed network that goes beyond the direct effect of disrupted auditory functions, on to language and communication development (Miron et al., 2016; Geva et al., 2017; Chen et al., 2019). Notably, individuals diagnosed with ASD display increased susceptibility not only to auditory processing deficits but to difficulties in other sensorial modalities, such as vision.

### Visual Processing and Gaze

The optic tract develops *via* a genetically driven regulation of axonal growth, navigation, and neuronal migration in the retinogeniculate pathway. The tract’s development also depends on endogenous and exogenous stimulation during the first years of life to secure and strengthen the synapses that refine the topographic map in the thalamic lateral geniculate nucleus and primary visual cortex (Graven, 2004). The more primal and fast to react dorsal visual stream is highly operational during the first months of life, relaying low-resolution data from the rods with increased sensitivity to changes in the exterior scenery (Hammarrenger et al., 2003; Bridge et al., 2016). The role of the superior colliculus (SC) in the dorsal stream has been recently highlighted, suggesting that in the neonatal period, this midbrain structure is pertinent for exercising focal oculomotor operations, receiving and integrating multimodal sensory inputs, and communicating with higher-order visual-neural configurations (Pitti et al., 2013; Jure, 2019). The SC is, thus, highly involved in rudimentary social behaviors, including the preference to fixate on human faces and the ability to detect and imitate emotion-resonating facial expressions (Jure, 2019). Neonatal experiences drive the SC to refine its ability to integrate inputs from diverse sensorial modalities in ways that expand social capabilities (Stein et al., 2014). Impairments in SC-contingent functions are found in populations with ASD.

An important line of evidence accentuating the major role of visual refinement through the SC for social-communication development is that congenital blindness is a significant risk factor for ASD, affecting approximately 50% of infants born without the ability to see (Jure et al., 2016). Ample evidence for the apparent vulnerability of the dorsal stream network is noted in a wide range of both genetic and acquired developmental disorders (Grinter et al., 2010; Braddick et al., 2011). With specific regard to ASD (Grinter et al., 2010), deficits in stabilizing visual fixation at 6–9 months were shown to predict social-communication problems at 36 months (Wass et al., 2015). These data suggest that deficits in apprehending the spatial grid and dynamic movements of objects (abilities rooted in the dorsal stream) have a major effect on the ability to execute contingent motor actions with social agents.

Notably, during the first year of life, the “fine-tuning” of the visual system for processing complex and socially charged stimuli is impaired in infants who are siblings of children with ASD (Zivan et al., 2021) and in those who are subsequently diagnosed with ASD (Zwaigenbaum et al., 2005; Elsabbagh et al., 2012; Jones and Klin, 2013). Later in development, abnormalities in social gaze patterns (Wegiel et al., 2013; Frazier et al., 2017) and other oculomotor functions (Johnson et al., 2016) are significantly associated with ASD. We suggest that the association between newborns’ visual processing indices, particularly the reactivity to highly salient, social, and moving stimuli, could serve as possible markers for a dorsal-colliculi deficiency in the BIAF and should be further investigated. Similar somatosensory processing dysfunctions should be further addressed.

### Gustatory and Olfaction Processing

Individuals diagnosed with ASD are more likely to have odors and tastes identification impairments (Bennetto et al., 2007; Boudjarane et al., 2017). Of specific interest to the BIAF is the trigeminal bottom-up olfactory pathway that innervates the nasal mucosa to execute protective respiratory reflexes in the presence of noxious odorants (Pérez de los Cobos Pallares et al., 2016). Operations of this pathway can be observed in newborns’ behavioral responses of disgust following exposure to unpleasant odors (Soussignan et al., 1997) and their autonomic regulation of breathing (Marlier et al., 2005). One of the primary odors for newborns is maternal odors during feeding. A meta-analysis found a negative association between maternal breastfeeding and ASD (Tseng et al., 2019); the authors interpreted the results by suggesting that breastfeeding has a moderating effect, but the involved mechanism is yet to be determined. Apart from the acknowledged importance of touch and emotional investment associated with breastfeeding, congenital deficits in olfactory, gustatory, and motor functions could add significantly to the accounts of both phenomena and to difficulties in the initiation of breastfeeding (Suberi et al., 2018). Taken together, these data suggest that impaired trigeminal reflexes in the newborn could be an additional BIAF early marker.

### Tactile-Motor Integration

Changes in responses to tactile stimulation have been acknowledged as a distinguishable feature of people with ASD (Wiggins et al., 2009; Foss-Feig et al., 2012; Balasco et al., 2020). Importantly, this network is rooted in the brainstem. The inferior olivary nucleus (ION) is an axial brainstem hub that receives multimodal sensory inputs, including tactile sensations. Through climbing excitatory fibers to the Purkinje cells, the ION enables the execution of coordinated motor actions (Wu et al., 2010; Ju et al., 2019).

Aberrant structuring of the ION, as found in populations with ASD (Rodier et al., 1996; Bailey et al., 1998), might restrain the valence of early life experiences, and the latency and proclivity to react *via* oscillations of efferent motor fibers (Arndt et al., 2005). Indeed, reduced tactile-motor reactivity at 12 months in the context of parent-child interaction was shown to be a risk factor for a subsequent diagnosis of ASD (Baranek, 1999). Given that collecting tactile information depends on perception and execution of movement, the findings suggest that the difficulties in tactile processing in children with ASD are intertwined with motor development. This notion is corroborated by the increased risk for impairments in motor functioning found in infants and toddlers who later develop autism (West, 2019). Taken together, these suggest that a deficit in ION-mediated tactile-motor rhythmicity might progress into a broader difficulty in the timing of communication and compromise social-communication efficacy in ASD.

### Sensorimotor Exchanges

A shortfall in synchronous sensorimotor communications with the world is almost intrinsic to the experience of children with ASD and their caregivers. It has profound implications on developmental outcomes in isolating the self from the social sphere, restricting exposure to familiar sensations, and limiting the sense of communicative agency (Delafield-Butt and Trevarthen, 2018).

Rhythmic communications, in which our senses swiftly grasp the exterior surrounding and contribute to it with our actions, are essential for building our sense of relatedness with the world and the people around us (Keller et al., 2014). The reviewed early life indices of sensorimotor integration suggest that early dysfunctions in brainstem systems impede the typical progression of the embodiment of social exchanges *via* alterations of the valence of sensorial inputs and the latency, vitality, and congruency of sensorimotor reactions. Importantly, several markers that could trace the full-blown ASD phenotype to brainstem-mediated abnormalities in newborns and infants were pinpointed. Their integration enables the development of an integrated BIAF.

## Integrating Individual Markers Into a Cohesive Brainstem-Informed Autism Framework

**FIGURE 1:**
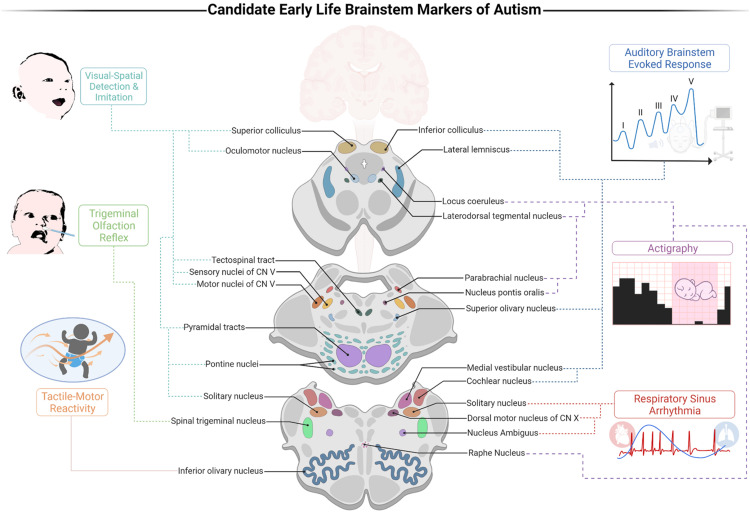
A proposed brainstem-informed autism framework (BIAF) screener for identification of early risk for autism spectrum disorders (ASD). Illustration of the links between the suggested markers and their brainstem substrates. Auditory processing is weighed by the auditory brainstem evoked response (ABR) test (specifically, a delayed V peak latency); DSW: near birth. Control of arousal is weighed by the cardiorespiratory index of respiratory sinus arrhythmia (RSA); developmental screening window (DSW): 0–12 months. Olfaction processing is weighed by the trigeminal olfaction reflex (disgust response following exposure to noxious odorants); DSW: 0–2 months. Sleep-wake regulation is weighed by actigraphy indices (*e.g.*, sleeping duration and sleep efficiency); DSW: 1–4 months. Visual processing and gaze are weighed by the abilities to detect and imitate emotional resonating facial expressions; DSW: 1–6 months. Tactile-motor integration is weighed by indices of response thresholds following tactile stimulation; DSW: 1–12 months. Created with BioRender.com.

It has been shown that the ABR alone has a noteworthy sensitivity for detecting infants who later develop ASD, with 70% accuracy (Miron et al., 2016). We suggest that integrating the individual markers points to the importance of a cohesive early life BIAF. The advent of such a framework offers advancements in our multidimensional understanding of autism. First, the BIAF proposes that the susceptibility to ASD develops during gestation and marks the neural networks that account for its early presentations, those that precede DSM symptoms oftentimes. Secondly, given the richness of the pathways traversing through the brainstem, the BIAF accounts for the heterogeneity in autism. Thirdly, the integrated BIAF may inform and promote the establishment of a clinical screener that will build upon prominent indices of brainstem functions to reach high sensitivity and reliability for weighing the risk for social development deficits. Such a screener could also ascertain the domain-specific impairments for each infant and reveal in which functions auxiliary support is necessitated (*e.g.*, auditory or tactile processing, autonomic-arousal reactivity, sleep hygiene, etc.). Accordingly, an early life BIAF screener could provide clinicians substantial gains vis-à-vis early detection of susceptible populations using electrophysiological indices (Figure 1, depicted on the right) along with behavioral ones (Figure 1, depicted on the left). The BIAF screener may carve new opportunities for customized interventions for the specific infant’s needs.

### Theoretical and Clinical Applications of the Brainstem-Informed Autism Framework

The BIAF has impactful applications for the scientific and clinical fields in the following key areas: (a) diagnostics, (b) prevention and moderation of symptoms, and (c) applicability. We suggest that these domains should be advanced in the following directions:

a. Diagnostics: An early life screener for detecting compromised brainstem functions should be implemented by assembling pertinent brainstem-mediated indices. A possible screener could consist of cardiorespiratory (*e.g.*, RSA), sleep-related (*e.g.*, actigraphy), and behavioral (*e.g.*, facial-emotional reactivity) indices. We suggest that such a screener should be further evaluated and could bring about remarkable prospects for early detection of susceptibility to ASD.
While detection of some of the proposed BIAF markers requires further research, the use of brainstem-mediated indices in the neonatal period can already enable pinpointing susceptible populations, as ABR protocols for the detection of early risk for autism are available (Miron et al., 2016). However, we suggest that adding additional BIAF indices may improve the sensitivity of early ASD detection compared to relying only on the ABR.
b. Prevention: Early detection of risk for ASD may enable referral to follow-up assessments and interventions at a sensitive period when the brain plasticity is exceptionally high. The opportunity of amending the pervasive changes in neural architecture has a high potential in moderating the protracted disturbances in brainstem functions, including cardiorespiratory reactivity, sleep-wake homeostasis, and sensorimotor development (Welch et al., 2015, 2020; Beebe et al., 2018).
c. Support, Intervention, and Vital Care: Recent notions highlight the pivotal role of calming regulatory interaction cycles between the child and the environment in shaping autonomic and social development (Ludwig and Welch, 2020), thus, expanding the role of parent-child interaction that is also central to our vertical hierarchical model (Geva and Feldman, 2008). These weighty notions of the *calming cycle theory* suggest that brainstem-mediated symptoms often associated with ASD should be viewed as treatable traits shaped in a dyadic context at sensitive stages of development. Assimilation of these ideas urges clinicians and scientists to conceptualize physio-emotional development as an open *co*-regulatory feedback system, including infants (and even fetuses) and their caregivers, rather than a process of a singular entity working in solitude (Ludwig and Welch, 2019). Such an approach suggests that the infant’s ability to regulate the autonomic activity for homeostasis, socioemotional and learning purposes is materialized in concert with the caregiver, as a dyad. We embrace this approach and suggest that aiding infant-caregiver dyads in preventing hyperexcitation and promoting tranquil dyadic experiences could be vital for moderating the risks posed by brainstem dysfunctions in susceptible infants. We further suggest that nurturing parents’ ability to mind and accurately attend to susceptible infants’ autonomic and sensorimotor cues is highly promising and calls for further evaluations in future BIAF interventions studies.
d. Applicability: Implementation of an early life BIAF screener for identifying infants with an increased risk for ASD could prove to be highly applicable and, in the long term, may aid in diminishing to some extent the related economic burden in several ways:
  i. The ABR is a cost-effective test and is already administered to millions of newborns worldwide. It could be easily recalibrated using specifically tailored protocols (Miron et al., 2016) to detect early risk for long-term social-communication deficits on large scales.
  ii. Developing a cohesive BIAF screener could reinforce the sensitivity of the ABR with additional brainstem-mediated behavioral, cardiorespiratory, arousal, and sleep-wake indices, hence, possibly transforming the prospect of early detection of ASD from theoretical to applicable. As time is of the essence, early identification of susceptible infants could provide us with better opportunities to amend the long-term developmental outcomes by referring them and their families to early interventions when brain plasticity is most receptive to modification– targeting primary brainstem-mediated neurobehavioral symptoms that are often associated with ASD.
  iii. Offering treatments compatible with the BIAF early in development is highly promising. Treatments that employ co-regulatory processes targeting brainstem-informed domains by fostering calming cycles carry the prospect of ameliorating to some extent the pervasiveness and suffering attributed to social-communication deficits throughout life (Geva and Feldman, 2008; Welch et al., 2015, 2020).

## Data Availability Statement

The original contributions presented in the study are included in the article/supplementary material, further inquiries can be directed to the corresponding author.

## Author Contributions

Both authors wrote the manuscript together and contributed equally to this article, and approved the submitted version.

## Conflict of Interest

The authors declare that the research was conducted in the absence of any commercial or financial relationships that could be construed as a potential conflict of interest.

## Publisher’s Note

All claims expressed in this article are solely those of the authors and do not necessarily represent those of their affiliated organizations, or those of the publisher, the editors and the reviewers. Any product that may be evaluated in this article, or claim that may be made by its manufacturer, is not guaranteed or endorsed by the publisher.
